# Human trophoblast stem cell self-renewal and differentiation: Role of decorin

**DOI:** 10.1038/s41598-018-27119-4

**Published:** 2018-06-12

**Authors:** Pinki Nandi, Hyobin Lim, Eloy Jose Torres-Garcia, Peeyush K. Lala

**Affiliations:** 1 Department of Anatomy and Cell Biology, Schulich School of Medicine and Dentistry, University of Western Ontario, London, Ontario Canada; 2 Department of Oncology, Schulich School of Medicine and Dentistry, University of Western Ontario, London, Ontario Canada; 3 Associate Scientist, Children’s Health Research Institute, Schulich School of Medicine and Dentistry, University of Western Ontario, London, Ontario Canada

## Abstract

The origin and regulation of stem cells sustaining trophoblast renewal in the human placenta remain unclear. Decorin, a leucine-rich proteoglycan restrains trophoblast proliferation, migration/invasiveness and endovascular differentiation, and local decorin overproduction is associated with preeclampsia (PE). Here, we tested the role of decorin in human trophoblast stem cell self-renewal and differentiation, using two models: an immortalized first trimester trophoblast cell line HTR-8/SVneo (HTR) and freshly isolated primary trophoblast (p-trophoblast) from early first trimester (6–9 weeks) placentas. Self-renewal capacity was measured by spheroid forming ability of single cells on ultra-low attachment plates for multiple generations. Markers of embryonic stem (ES) cells, trophoblast stem (TS) cells and trophoblast were used to identify stem cell hierarchy. Differentiation markers for syncytial and extravillous (EVT) pathways were employed to identify differentiated cells. Bewo cells were additionally used to explore DCN effects on syncytialization. Results reveal that the incidence of spheroid forming stem-like cells was 13–15% in HTR and 0.1–0.4%, in early first trimester p-trophoblast, including a stem cell hierarchy of two populations of ES and TS-like cells. DCN restrained ES cell self-renewal, promoted ES to TS transition and maintenance of TS cell stem-ness, but inhibited TS cell differentiation into both syncytial and EVT pathways.

## Introduction

Embryonic trophectoderm, the precursor of trophoblast cells of the placenta is the first epithelium appearing during mammalian development. Trophoblast stem cell maintenance and differentiation pathways have been well-defined in the mouse including establishment of trophoblast stem cell lines from the mouse blastocyst^1,2^. The stem cells have been categorized into embryonic stem (ES)-like and more committed trophoblast stem (TS)-like cells with distinct ES and TS cell markers. For a long time, it has not been possible to establish human trophoblast stem cell lines from the preimplantation blastocyst, and the source of stem cells that sustains trophoblast growth, renewal and differentiation in the human placenta still remains poorly characterized. It was reported that chorionic mesenchyme serves as a niche for human trophoblast stem cells^3^. The authors isolated cells expressing pluripotency markers from first and second trimester chorion, and grew them in the presence of FGF and inhibitors of the activin/nodal pathway to obtain self-replicating cells which gave rise to both syncytiotrophoblast (STB) and cytotrophoblst (CTB) with invasive phenotype simulating extravillous trophoblast (EVT). It is likely that chorionic mesenchyme is a source of pluripotent stem cells which were induced to form trophoblast *in vitro*. Similarly human embryonic stem cells lines (hESC) have also been successfully manipulated *in vitro* with bone morphogenetic protein (BMP) 4 treatment to obtain cells with STB and EVT phenotype^4–7^.The latter authors^7^ showed that hESCs after exposure to BMP4 and two small compounds, an activin A signaling inhibitor and a FGF2 signaling inhibitor (BMP4/A83–01/PD173074; BAP) commit to trophoblast lineages identified by markers of CTB and STB. They proposed that the STB generated from the differentiated hESC (hESC-d) represents the primitive invasive syncytium encountered in early pregnancy prior to the development of the chorionic villi. This hypothesis was further substantiated by a global and unbiased analysis of previously published transcriptomic profiles for hESC-d, showing that they lack a mesoderm signature and is a subtype of placental cells unlike those present at term, but akin to an invasive syncytium^7^. Whether pluripotent stem cells residing outside the confines of the chorionic villi serve as a source of trophoblast progenitors within the villi remain controversial. Numerous studies suggest that the source of human trophoblast stem cells lies in the CTB layer of the chorionic villi in the post-implantation placenta^6,8,9^. Indeed, very recently Okae *et al*.^10^ succeeded in establishment of long term cultures of human cytotrophoblast cells from early first trimester placentas and human blastocyst by activation of Wnt and EGF and inhibition of TGF-β, histone deacetylase, and Rho-associated protein kinase^10^.

The human placenta serves multiple functions essential for fetal growth and survival. These functions are provided by two distinct trophoblast cell classes: villous and extravillous trophoblast. Both of them originate from the bi-potent progenitors contained within the CTB layer of the chorionic villi^11^. Villous STB layer lining the maternal blood sinusoids, which arise by CTB cell fusion, are primarily engaged in absorptive, exchange and endocrine functions. EVT cells arise from the CTB as highly migratory cell columns which proliferate at the base of the anchoring villi and invade uterine decidua. Some invade the spiral arterioles by adopting an endothelial phenotype and remodel them from muscular, high-resistance, low-flow tubes into non-muscular, low-resistance, high-flow tubes that permit adequate flow of maternal arterial blood to nourish the fetus^12,13^. A compromised EVT invasion, endovascular differentiation and uterine arterial remodeling are implicated in the pathogenesis of preeclampsia (PE) in the mother and a subset of growth restriction in the fetus (FGR)^12,13^.

Molecular mechanisms regulating human trophoblast stem cell self-renewal and differentiation into STB and EVT lineages remain poorly understood^14–16^. Many epithelial tissues both during embryonic development and post-natal life contain transiently amplifying primitive progenitors which give rise to committed progenitor pools to replenish cells in the differentiation pathways^17^. Whether the CTB layer of the early gestational human placenta identified as the source of TS cells^10^ contains more than one progenitor cell class remains to be investigated.

An immortalized first trimester human trophoblast cell line HTR-8/SVneo (HTR) developed by us many years ago^18^ has been shown to contain bi-potent trophoblast progenitors. This was demonstrated by different approaches: an enrichment for α6β4 integrin expressing cells^19^ showing increased clonogenicity, isolation of a very minor “side population” capable of excluding the Hoschest dye^20^ and hanging drop preparations containing ~3000 cells/drop, which gave rise to “embryoid-like bodies or spheroids” with stem cell markers^21^. Whether such spheroids were clonal in origin, i.e, resulting from self-renewal of stem cells remains uncertain. Stem cells in neural tissues^22^ and the mammary gland^23^ with potentials for differentiating into several progenitor cell populations have been identified by their ability to form spheroids (neurospheres and mammospheres) when grown on ultra-low attachment (ULA) plates. Tumor biologists have exploited this technique to identify tumor-perpetuating stem-like cells (SLC) growing as spheroids (tumorspheres)^24^. This approach had been utilized by our laboratory to explore molecular mechanisms in SLC regulation in human breast cancer^25^. Spheroid-forming ability allows one to identify the incidence of stem cells by limiting dilution, as well as their ability for sustained self-renewal by repeated passages of the spheroid-derived cells. In the present paper, we have applied this method to HTR cells as well as primary trophoblast cells (p-trophoblasts) isolated from early first trimester human placentas to examine self-renewal and identify putative embryonic stem (ES) and trophoblast stem (TS) cell markers in cells within the spheroids. This approach was then utilized to explore the roles of a placenta-derived molecule decorin (DCN). Bewo trophoblast cells were additionally used to examine the role of DCN in syncytial differentiation.

DCN is a small leucine-rich, TGF- β binding proteoglycan produced by several stromal cell classes e.g., dermal fibroblasts, chondrocytes, chorionic villus mesenchymal cells in the placenta and decidual cells in the pregnant endometrium^26,27^.We discovered that DCN is co-localized with TGF- β in the decidual ECM^27^, playing a unique role in utero-placental homeostasis. DCN was shown to restrain trophoblast proliferation, migration and invasion, independent of TGF- β^28^ by differential binding to multiple tyrosine kinase receptors EGFR, IGFR-2 and VEGFR-2^29^. VEGFR-2 binding was localized to a 12 amino acid span of the leucine-rich-repeat (LRR) 5 domain of DCN^30^, which blocked endovascular differentiation of the EVT^31^. Furthermore, DCN overproduction by the decidua was shown to be causally associated with PE, by compromising trophoblast invasion, endovascular differentiation and uterine arterial remodeling^32,33^. We also found that elevated DCN level in maternal blood predates PE, serving as a potentially predictive biomarker for PE^32^.

DCN was reported to control nephron progenitor cell differentiation^34^ in a paracrine manner and shown to be a constituent of the ECM that maintained stem-ness in bone marrow progenitor cells^35^. Weber *et al*.^36^ reported unique trophoblast stem cell and pluripotency marker staining patterns in the human placentas depending on gestational age and placenta-associated pregnancy complications. They suggested an arrested stem cell differentiation in PE and FGR. We have reported that DCN over-activity at the fetal-maternal interface plays role in the pathogenesis of PE^32,33^. Furthermore, DCN was shown to be a blood biomarker for PE^32^ and possibly idiopathic FGR^37^. For these reasons, present study was designed to explore the roles of DCN in trophoblast stem cell self-renewal and differentiation. Our results indicate that DCN restrains self-renewal of ES-like cells, promotes their transition into and maintenance of TS-like progenitors, and inhibits TS cell differentiation into STB and EVT pathways.

## Results

Stem cells, by definition, have the capacity for both self-renewal and differentiation into different lineages. In the case of trophoblast stem cells, the well-known differentiation pathways are formation of STB and EVT both having their distinctive markers. In addition, staining for cytokeratin 7 should identify them as trophoblast rather than mesenchymal cells. We evaluated the self-renewal capacity of both HTR and primary trophoblast cells by the ability of single cells to form spheroids for successive generations. Expression of ES and TS-like markers were evaluated at the level of mRNA using qPCR and at the protein level by immunofluorescence. Differentiation capacity was examined by marker expression for both STB and EVT pathways.

### Formation of spheroids by HTR-8/SVneo cells for multiple generations

**Figure 1:**
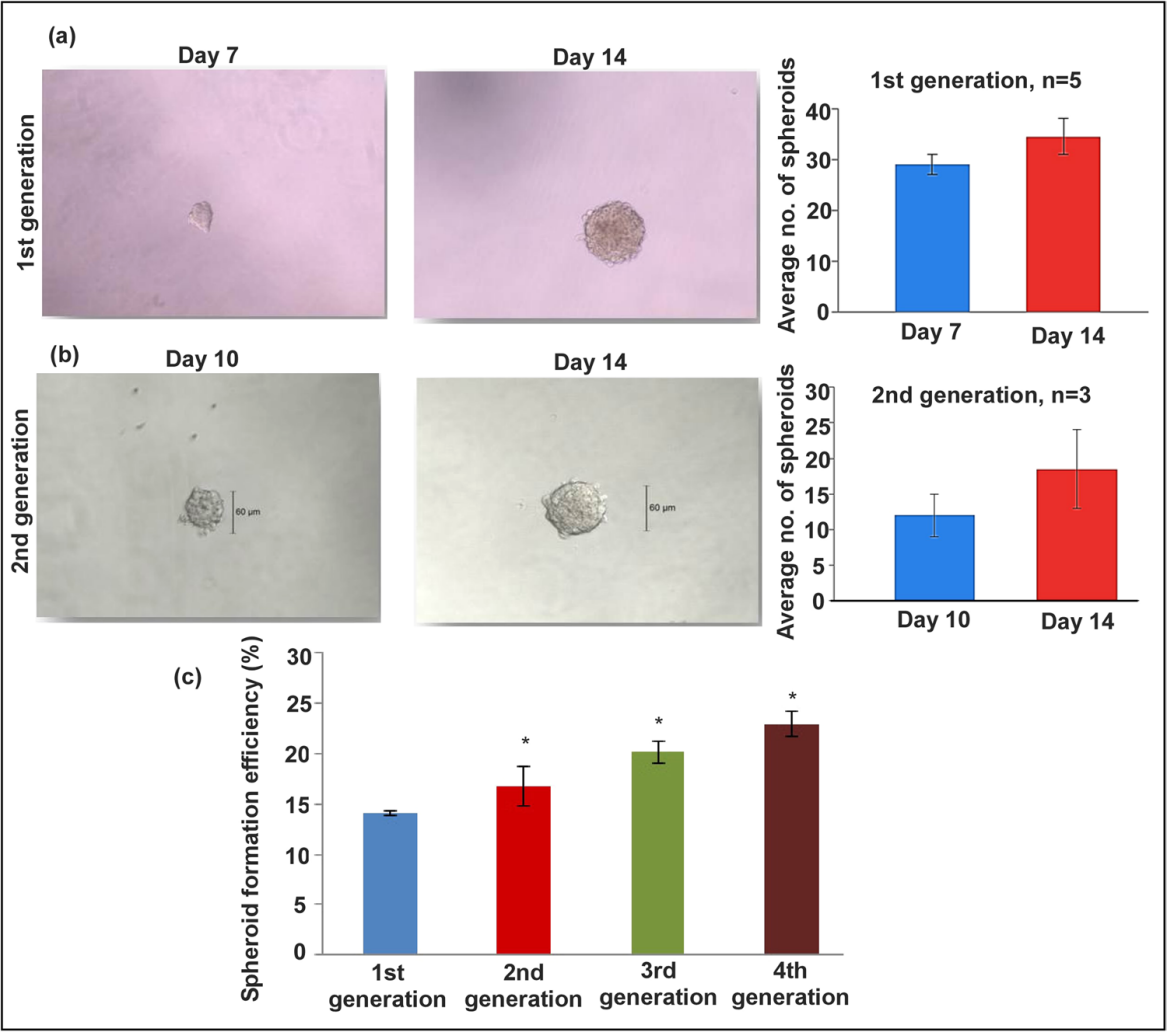
HTR cells grow as spheroids for multiple generations. HTR cells started to form spheroids on 96 well ULA plates, (5 cells/well) on day 3 and grew in numbers and sizes until day 10. (**a**) Shows the spheroid morphology and numbers on days 7 and 14 (n = 5). (**b**) Shows spheroid formation in the second generation (n = 3) and c shows the spheroid forming efficiency (%) for both generations. *Indicates p < 0.05.

HTR cells (without further selection for any markers or physical properties) formed spheroids on ULA plate (96 well, 5 cells plated /well) as early as on day 3 and grew in numbers and sizes until day 10. After 14 days, cells in the core of the spheroids showed cell death. Figure 1a shows the spheroid morphology and numbers on day 7 and 14. Figure 1b shows similar data in the second generation spheroids grown after dispersion of cells from first generation spheroids at 7–9 days of growth. Figure 1c shows the spheroid forming efficiency (%) for four successive generations with a small but significant increase in each generation. These data reveal the presence of stem like cells with capacity for self-renewal in the HTR cell line which is immortalized but not tumorigenic.

### Changes in ES and TS marker expression in HTR cells during spheroid formation

**Figure 2:**
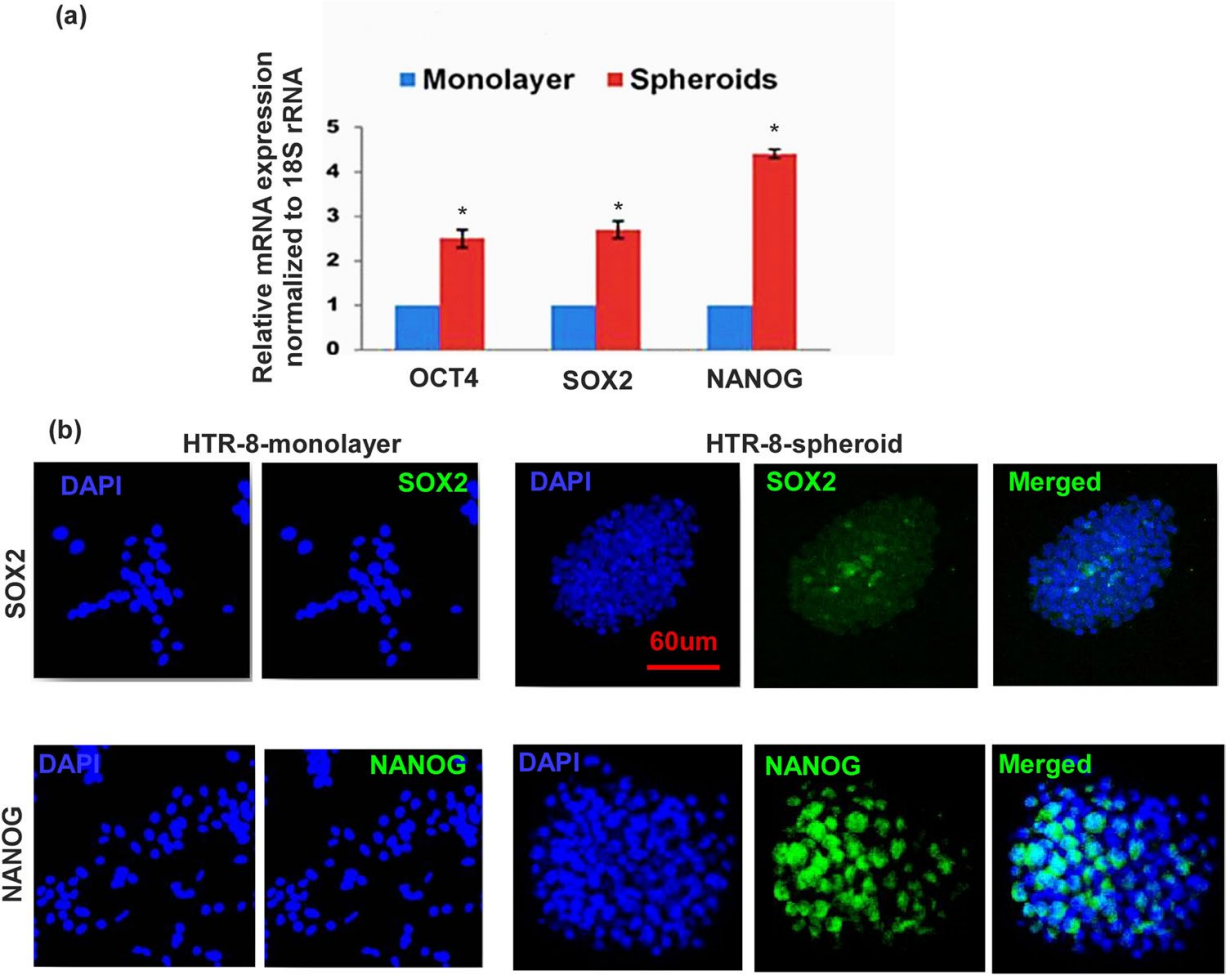
HTR cells grown as spheroids reveal marked increase in ES markers compared to monolayers. (**a**) Shows mRNA expression of ES markers OCT4 (C_t_ = 23.1), SOX2 (C_t_ = 22.1), NANOG (C_t_ = 25.2),) in HTR cells grown as spheroids as compared to monolayers on plastic surface (n = 5, *indicates p < 0.05). (**b**) Shows immunostaining for SOX2 and NANOG under the same conditions. Representative images are selected from 5 different experiments.

**Figure 3:**
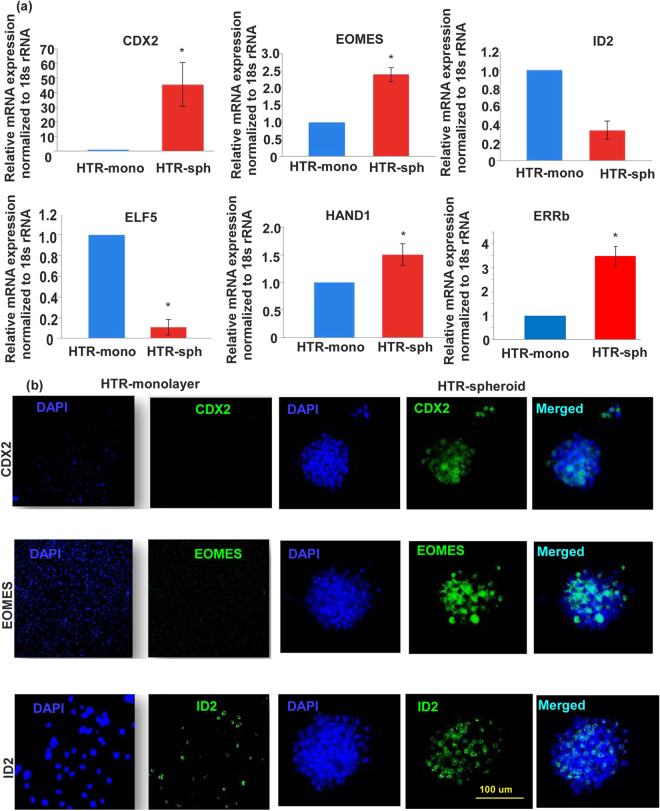
HTR cells grown as spheroids reveals marked increase in TS markers compared to monolayers. (**a**) Shows mRNA expression of TS markers CDX2 (C_t_ = 26.31), EOMES (C_t_ = 25.68), ID2 (C_t_ = 27.2), ELF5(C_t_ = 33.1), HAND1(C_t_ = 27.4), ESRRb (C_t_ = 32.6) in HTR spheroids compared to monolayers on plastic surface (n = 5, *indicates p < 0.05). (**b**) Shows representative images of immunostaining for CDX2, EOMES and ID2 under the same conditions.

ES-like markers (OCT4 SOX2, NANOG) and TS-like markers (CDX2, ELF5, EOMES, ESRR β, HAND1, ID2) have been well defined in the mouse^2,8^. Some of these markers have also been identified in human placenta^8^. Here utilizing the same markers we found significant increase in mRNA expression of the above markers (with exception of ELF5 and ID2) in HTR cells grown as spheroids for 7–9 days as compared to monolayers on plastic surface (Figs 2a and 3a). Immunostaining of HTR monolayer and spheroids for some of the selected makers (SOX2, NANOG, Fig. 2b) and (CDX2, EOMES, ID2, Fig. 3b) revealed absence or paucity of stained cells in the monolayers, while a high incidence of stained cells were found within the spheroids (Figs 2b and 3b; negative controls provided in Supplementary Figure 1a and b). The TS marker expression was also confirmed in second generation spheroid (data not shown). These findings clearly indicate that spheroid-forming cells represent stem-like cells during trophoblast development. Interestingly when we did double immunostaining for ES and TS markers in the same spheroid, so far we found that individual spheroids (n = 12) expressed either ES or TS marker. It is likely that the double marker containing cells (in transition from ES to TS cells) are a very small minority so that we could not detect them.

### Differentiation potential of cells within HTR-spheroid and spheroid derived cells

**Figure 4:**
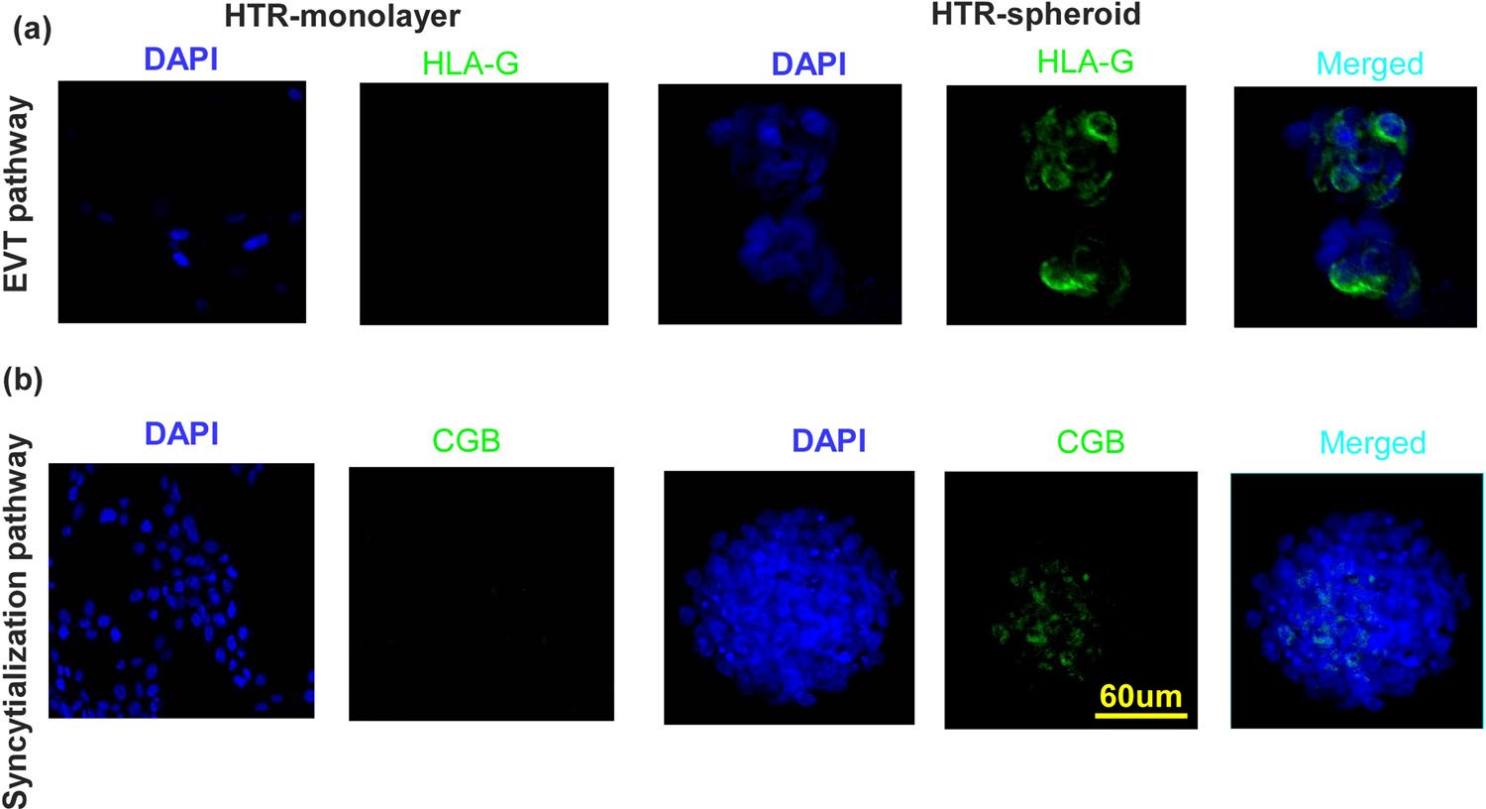
HTR spheroids contain bi-potential progenitors for differentiation into EVT and syncytialization pathways. HTR cells grown as monolayers did not stain for HLA-G protein while spheroid derived cell clumps cultured overnight on plastic show HLA-G protein expression (**a**). Similarly there was no immunostaining for CGB on monolayer cells grown on plastic whereas some cells within the spheroid express CGB (**b**).

HLA-G is a selective marker expressed by EVT cells^38^. We have earlier shown that cell surface HLA-G expression is modulated in the HTR cell line depending on the passage and growth conditions^30^. When grown on differentiation inducing matrix, laminin or matrigel, they always express HLA-G protein^39^. In the present study HTR monolayer cells grown on plastic did not stain for HLA-G. On the other hand spheroid derived cells cultured overnight on plastic show HLA-G protein expression (Fig. 4a shown by immunostaining) indicating differentiation into the EVT pathway without any differentiation inducing matrix. Similarly there was no immunostaining for CGB (villous pathway markers) on monolayer cells grown on plastic whereas some cells within the spheroid express CGB indicating differentiated progenies (Fig. 4b). We also investigated the co-expression of ES, TS markers along with differentiation markers by qRT-PCR. Our data reveal expression of ES (SOX2, NANOG) TS (CDX2, EOMES) and differentiation (HLA-G and CGB) markers in a population of pooled spheroids (Supplementary Figure 2).

### Effects of DCN on HTR-8 spheroid formation and marker expression

**Figure 5:**
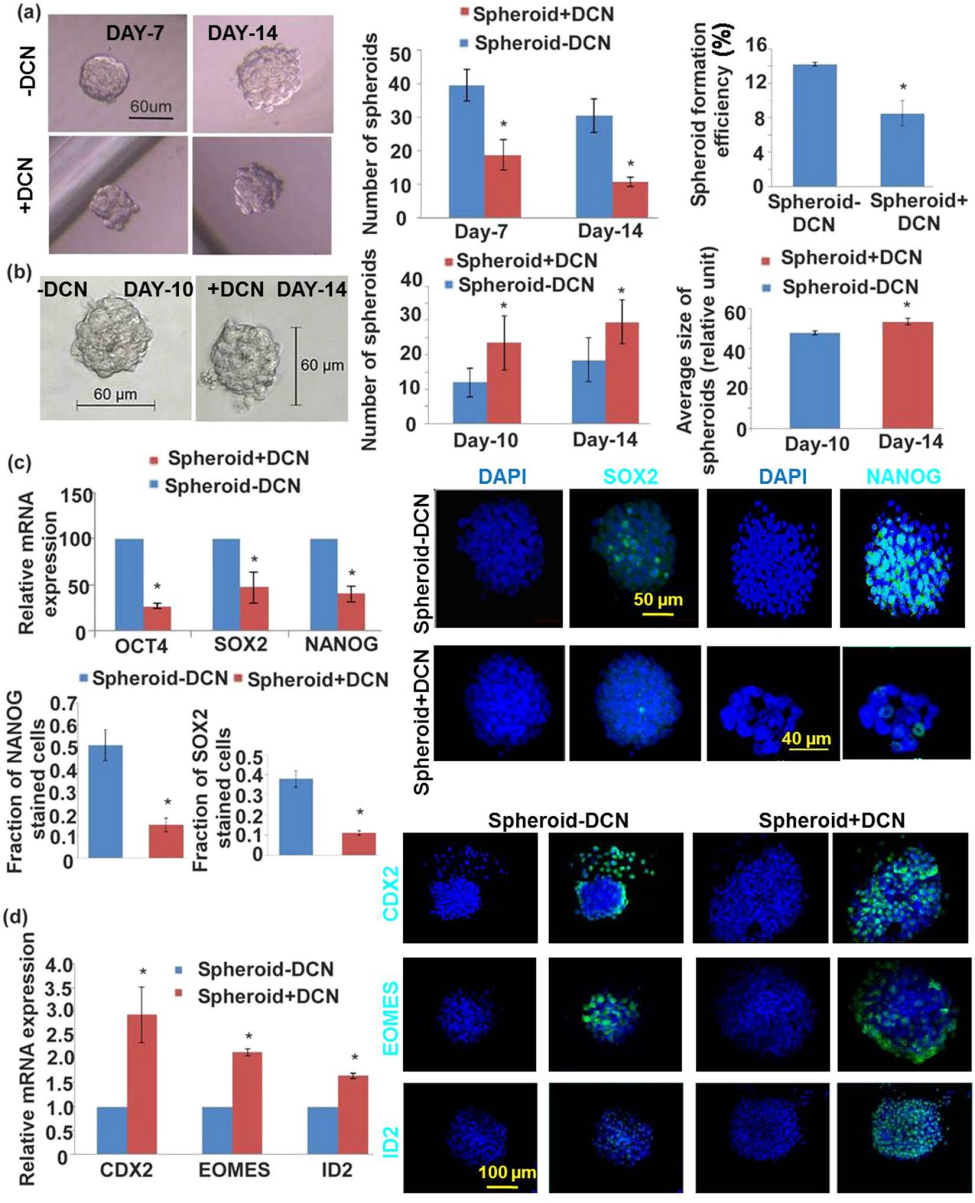
DCN inhibits HTR-8 spheroid formation and differentially regulates ES and TS marker expression. (**a**) Shows DCN effects (250 nM, having no effect on cell viability), on spheroid numbers and spheroid forming efficiency during the first generation. (**b**) Shows sizes and number of second generation spheroids resulting from DCN-treated first generation spheroids. Significantly reduced mRNA expression of ES cell markers (OCT4, SOX2, NANOG, **c**) but increased expression of some TS cell markers (CDX2, EOMES and ID2, **d**) was noted in first generation spheroid in presence of DCN (n = 5, *indicates p < 0.05). Representative immunostaining of spheroids revealed a reduction in ES marker bearing cells (**c**) with a concomitant increase in TS marker bearing cells (**d**).

In the presence of exogenous DCN (250 nM, having no effect on cell viability), number of spheroids and spheroid forming efficiency during the first generation were significantly reduced (Fig. 5a). There was no significant effect of DCN on spheroid size. Second generation spheroids resulting from DCN-treated first generation spheroids showed increase in sizes and numbers compared to those from DCN-untreated first generation spheroids (Fig. 5b). Spheroid forming efficiency increased in a manner similar to the increase in spheroid number (data not shown). These results indicate that exposure of cells to DCN reduces their self-renewal ability in the short term but promotes their self-renewal in subsequent generations. Expression of ES and TS cell markers were investigated in order to decipher the roles of DCN on ES and TS cell properties. Presence of DCN (250 nM) significantly reduced the mRNA expression of ES cell markers (OCT4, SOX2, NANOG, Fig. 5c) but increased the expression of some TS cell markers (CDX2, EOMES and ID2, Fig. 5d) in first generation spheroid-derived cells. Similarly, immunostaining of spheroids revealed a reduction in ES marker-bearing cells (Fig. 5c) with a concomitant increase in TS marker-bearing cells (Fig. 5d). These findings reveal that DCN inhibits ES cell self -renewal but promotes TS cell self - renewal and commitment.

### Effects of DCN on differentiation of HTR spheroid derived cells

**Figure 6:**
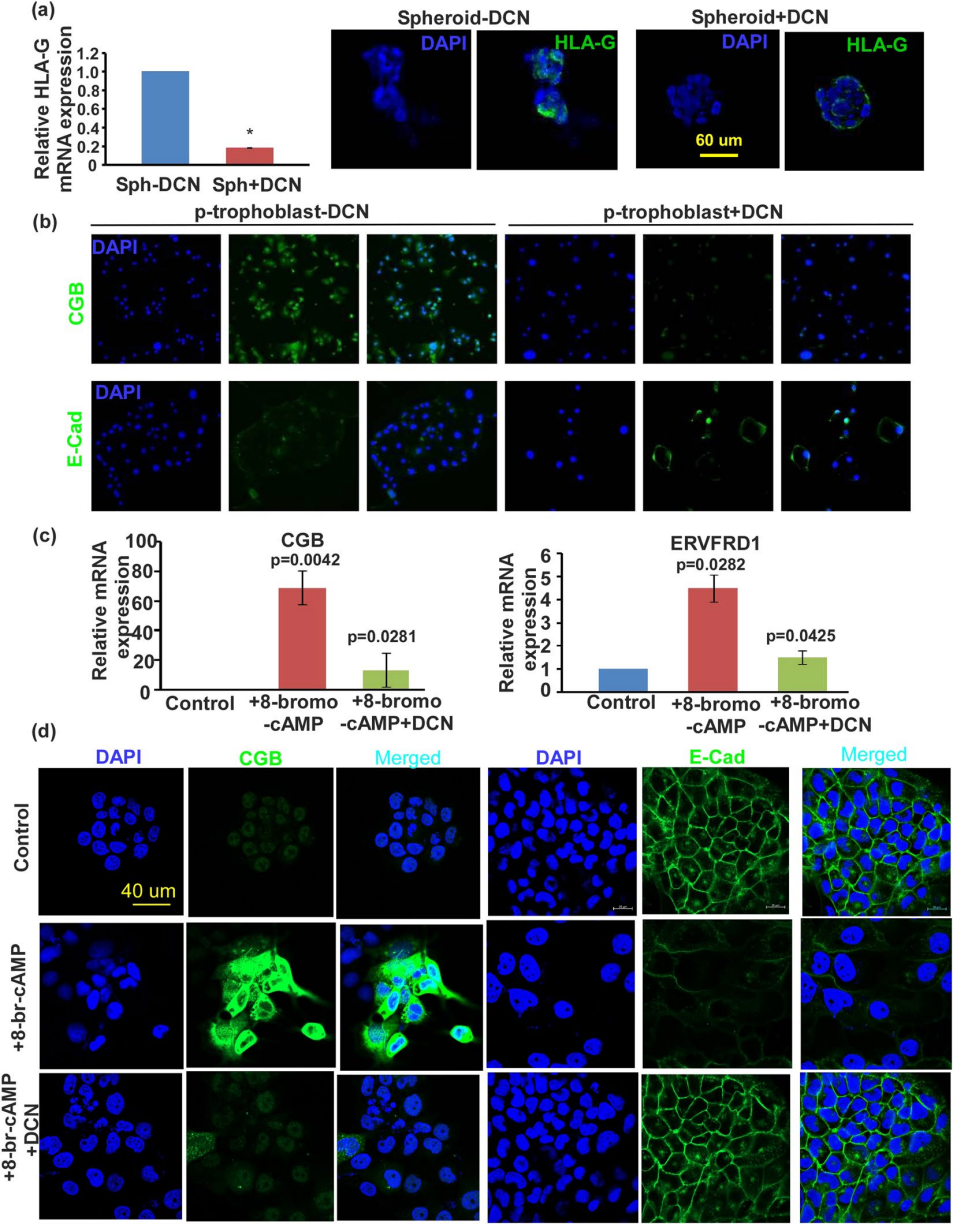
DCN inhibits differentiation of trophoblast cells. (**a**) Shows DCN effects on HLA-G (EVT marker) mRNA (left panel, by qPCR) and protein expression (immunostaining) in HTR-spheroid derived cells. (**b**) Shows representative images of DCN effects on syncytialization markers (loss of junctional E-cadherin and stimulation of CGB expression) by immunostaining in p-trophoblast cells grown as monolayer. (**c**) Shows DCN effects on mRNA expression (of CGB and Syncyin) for 8-bromo cAMP induced syncytialization in Bewo cells (n = 5, *indicates p < 0.05). (**d**) Shows representative images of DCN effects on syncytialization markers (loss of junctional E-cadherin and stimulation of CGB expression) by immunostaining in Bewo cells.

DCN significantly decreased HLA-G mRNA (qPCR) and protein (Immunofluorescence) expression in HTR-spheroid derived cells indicating a suppression of differentiation into EVT pathway. However 8-bromo-cAMP treatment (a widely used inducer of syncytialization) failed to induce the HTR-spheroid derived cells to differentiate further into the villous pathway when cultured on plastic surface (evidenced by absence of immunostaining for CGB and GCM1 marker, data not shown). Hence we resorted to primary trophoblast cultured in monolayer to syncytialize spontaneously to examine the effect of DCN (Fig. 6b). Immunostaining data for CGB and E-cadherin reveals that DCN inhibits the syncytialization pathway. In addition, we utilized the well-known Bewo cell model to explore DCN effects (Fig. 6c,d). In the presence of 8-bromo cAMP, cells expressed high levels of CGB and syncytin identified with qPCR (Fig. 6c) and immunostaining (Fig. 6d). The 8-bromo cAMP treated cells lost cell junction associated E-cadherin. DCN treatment partially reversed all of these effects of cAMP on syncytialization.

### Spheroid forming ability of cytotrophoblast cells isolated from first trimester human placenta

**Figure 7:**
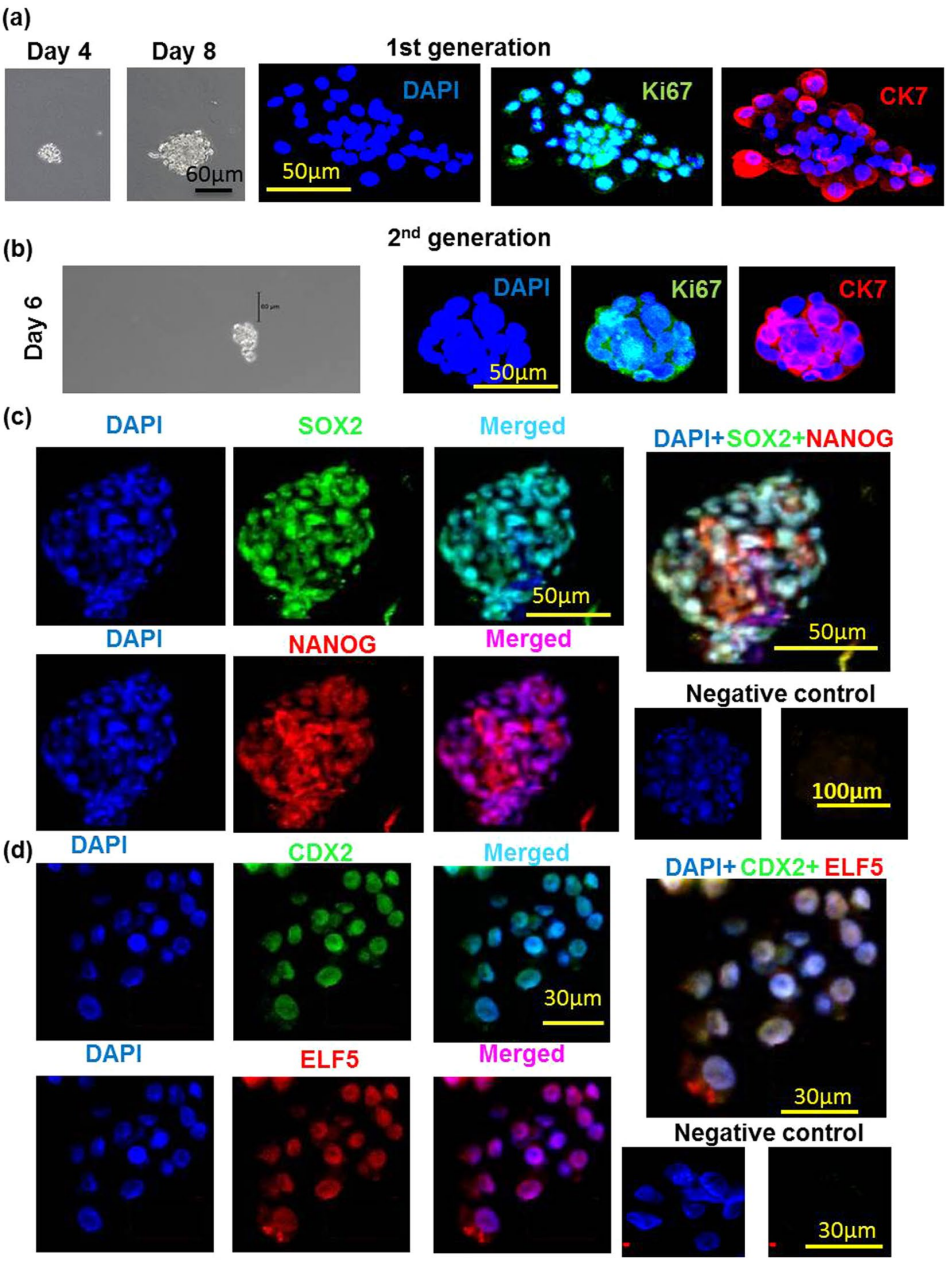
Human first trimester cytotrophoblast cells form spheroids which contain proliferative cells and express both ES and TS cell markers. (**a**) Shows spheroids formed on days 4 and 8 by freshly isolated p-trophoblast and co-immunostaining for CK7 (trophoblast marker) and Ki67 (proliferation marker) in the first generation. Cells dispersed from the first generation spheroids also produced spheroids during the second generation and stained positive for CK7 and Ki67 (**b**). (**c**) Shows co-expression of ES markers OCT4, NANOG in cells within the p-trophoblast spheroids. (**d**) Shows co-expression of TS makers CDX2, ELF5 in cells within p-trophoblast spheroids.

Freshly isolated p-trophoblast from 6–9 weeks human chorionic villi resulted in 95–98% purity (CK7 staining, data not shown) and ~99% viability (Trypan blue dye exclusion). Spheroid forming ability of p-trophoblast is shown in Fig. 7a (shown for day 4) for first generation spheroids. Distinct spheroid formation was observed on day 3 with an increase in numbers and sizes of spheroids until day 10 (shown for day 8). Based on the use of 12 first trimester (6–9 week) placentas at different times, the spheroid forming efficiency of the primary CTB in the first generation was found to range between 0.1–0.4 percent, the efficiency declining with the gestational age. Trophoblast cells isolated from placentas retrieved at 10–12 weeks (n = 4) hardly formed any spheroids. Cells dispersed from the first generation spheroids also produced spheroids during the second generation (Fig. 7b). Spheroid forming efficiency for the second generation could not be precisely determined because of low number of cells isolated from the first generation spheroids.

### Proliferative capacity of cells within first and second generation spheroids

p**-**Cytotrophoblast cells quickly lose their proliferative capacity after isolation and spontaneously syncytialize on traditional plastic surface. To investigate the proliferative capacity of cells within the spheroids, Ki67 marker staining was employed. CK7 staining was used to confirm trophoblast identity. Figure 7a shows co-staining of both markers in almost every cell in first generation spheroids. While CK7 staining is maintained in almost every cells in the second generation spheroids, the incidence of Ki67 stained cells showed some decline (Fig. 7b). These data clearly revealed that p-CTB cells can proliferate beyond 2 weeks in spheroid cultures.

### Expression of ES and TS cell markers in p-trophoblast spheroids

We found that cells within the p-trophoblast spheroids express ES markers SOX2, NANOG (Fig. 7c) and TS makers CDX2, ELF5 as revealed by immunostaining (Fig. 7d). So far we have not been able to identify any spheroid expressing both ES and TS markers from the limited number of spheroid tested (n = 7) subjected to double immunostaining.

### Effect of DCN on p-trophoblast spheroid

**Figure 8:**
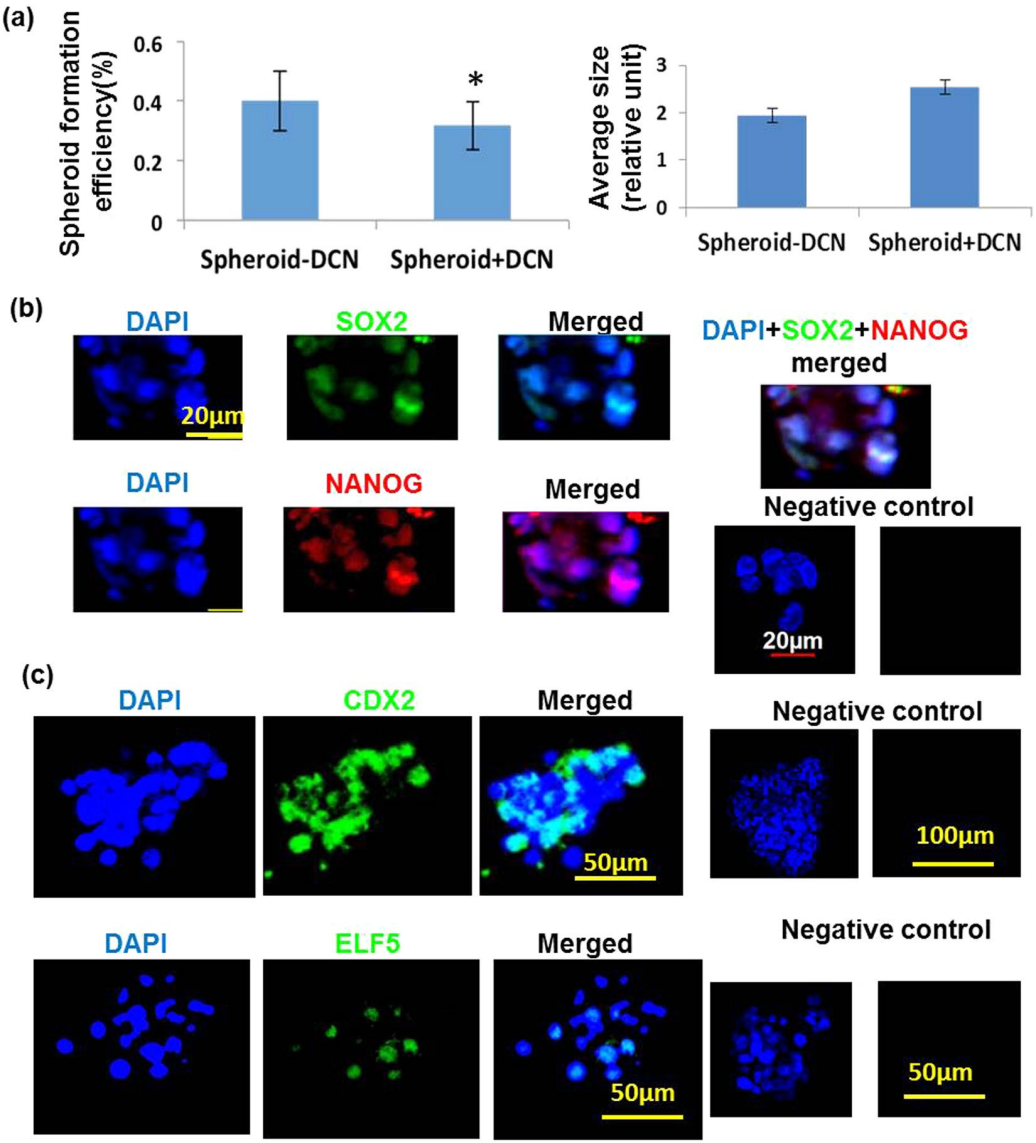
Decorin reduces spheroid forming efficiency of first trimester cytotrophoblast cells. (**a**) Shows that DCN reduced spheroid forming efficiency (n = 5, *indicates p < 0.05) with a trend in increased spheroid sizes (not significant). Both ES (SOX2 and NANOG, **b**) and TS cell markers (CDX2 and ELF5, **c**) were also present in DCN treated spheroids as revealed by immunostaining.

DCN treatment appeared to reduce spheroid forming efficiency with a trend in increased spheroid sizes which were not significant (Fig. 8a). Both ES (Fig. 8b) and TS cell markers (Fig. 8c) were also present in DCN treated spheroids but could not be quantified precisely due to limited number of spheroids.

### Schema of proposed DCN action on stem cell hierarchy

**Figure 9:**
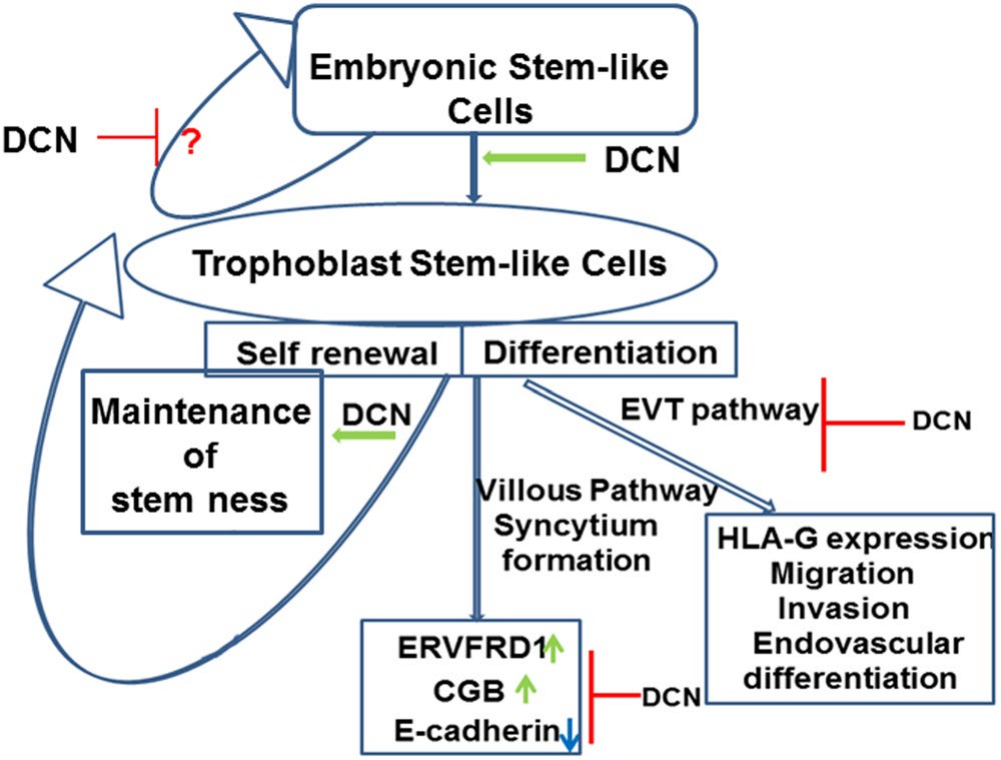
Schema of proposed DCN action on stem cell hierarchy. Figure 9 summarizes the DCN effects on stem cell self-renewal and differentiation: inhibition of self-renewal of ES-like cells (reduced spheroid numbers); promotion of ES differentiation to TS-like cells (increased TS markers); promotion of self-renewal of TS-like cells (increased second generation spheroid forming efficiency); inhibition of differentiation of TS-like cells to villous (syncytium formation) and EVT pathways (marker expression).

Figure 9 summarizes the proposed DCN effects to best fit the results**:** inhibition of self-renewal of ES-like cells (reduced first generation spheroid forming efficiency); promotion of ES differentiation to TS-like cells (increased TS markers); promotion of self-renewal of TS-like cells (increased second generation spheroid forming efficiency); inhibition of differentiation of TS-like cells to villous (syncytium formation) and EVT pathways (marker expression).

## Discussion

Until very recently (2018) no good model was available to study the dual properties of human trophoblast stem cells i.e., self-renewal and differentiation at the same time. Okae *et al*.^10^ reported long term culture of first trimester human trophoblast cells in special culture conditions which differentiated to both STB and EVT pathways. However, they failed to derive TS cells from single CTB cells. Our study for the first time shows that spheroid formation from single cells on ULA plate is a valuable approach to examine both parameters for the human trophoblast. This was achieved using an immortalized and non-tumorigenic first trimester trophoblast cell line HTR/SVneo produced in our laboratory many years ago^18^ and used worldwide as an *in vitro* model to study trophoblast biology. We believe that during the immortalization procedure a small subset of stem-like cells in the primary trophoblast (HTR-8) was immortalized to maintain their stem-ness at a much higher efficiency (40–100 fold) than the primary CTB, as estimated from a comparison of the spheroid forming efficiency of the p-trophoblast with that of HTR. Because of this high efficiency in spheroid formation in HTR cells we could dissect self-renewal and differentiation capacity of the cells and the effects of DCN on both the stem cell attributes. We also validated the self-renewal and differentiation capacities in p-trophoblast derived spheroids. Our findings of a rapid decline of spheroid-forming efficiency in p-trophoblast with gestational age during the first trimester is supportive of the report by Okae *et al*.^10^ of their inability to derive trophoblast stem cell lines from older gestational age placentas.

Present study using both HTR and p-trophoblast cells shows that growing cells in monolayer on plastic surfaces revealed paucity of ES and TS markers, quantified at the mRNA level and identified at the cellular level by immunostaining. On the other hand, growing cells as spheroids significantly increased both ES and TS cell markers at the mRNA and cellular level in HTR-spheroids. This was also shown at the cellular level in p-trophoblast spheroids. Ability of spheroid derived cells to grow during a second generation at a higher efficiency support the contention that spheroid forming cells are stem-like cells. Presence of both ES and TS-like cells suggests that there are more than one stem cell populations within the human trophoblast stem cell hierarchy similar to that shown in case of mouse trophoblast stem cells^40^. Our data are consistent with very short ES → TS transition, since we could not identify both ES and TS marker -bearing cells with dual immunostaining of limited number of spheroids.

Decorin is a product of both fetal mesenchymal cells within the placenta and decidual cells in the endometrium. Since DCN can be secreted in a soluble form and transported to the CTB layer from the fetal mesenchymal cells, it is logical to suggest that it can exert a physiological role in trophoblast progenitor self-renewal and differentiation. Effects of exogenous decorin could be fully analyzed in HTR spheroids and only partially validated in p-trophoblast spheroids due to very low spheroid forming efficiency in the latter case. A reduction in the spheroid forming efficiency in DCN treated cells during first generation but an increase in the second generation indicated that DCN inhibited the self-renewal capacity in the short term but promoted self-renewal in longer term. A reduction in ES marker expression during the first generation suggested DCN mediated inhibition of ES cell renewal. A concomitant increase in TS cell markers suggest ES-TS conversion and commitment to TS cells. Increase in spheroid forming efficiency during the second generation reinforce the concept that DCN promoted TS cell self-renewal.

Earlier studies using our HTR-8/SVneo cell line have provided the evidence of the presence of trophoblast progenitor (putative stem) cells by using different approaches. Takao *et al*.^20^ reported that a minor “side population” isolated by exclusion of Hoechst dye had long term proliferation and differentiation capacity. They also expressed the TS cell marker ID2 at high level^41^. Weber *et al*.^21^ used hanging drop preparations of ~3000 cells/drop to show spheroid formation and their ability to grow for multiple passages. They also showed expression of some ES and TS cell markers. The spheroids derived from these cells might have resulted from cell aggregation rather than clonal growth. Our studies for the first time using spheroid formation on ULA plate reveal their capacity for clonal expansion by single cells and expression of both ES and TS cell markers. Interestingly, very recently another laboratory utilized HTR-SVneo cells in spheroid assays to provide evidence of the presence of cells bearing ES cell markers (OCT4, SOX2 and NANOG) within the spheroids grown on ULA plates and their alterations in doxo-rubicin resistance^42^. We suggest that this method allows us to measure self-renewal capacity of cells by limiting dilution (currently to 5 cells/per well) and from single cells.

The role of DCN in trophoblast stem cell functions has never been explored. DCN was reported to control nephron progenitor cell differentiation^34^. These authors found that *DCN* gene was repressed by FOXD1 transcription factor in cortical interstitial cells and that genetic inactivation of *DCN* partially rescued the failure of progenitor cell differentiation in the FOXD1 null cells. Also, DCN was shown to be a component of the ECM derived from mouse bone marrow which maintained stem-ness in bone marrow progenitor cells^35^. Interestingly in the microarray analysis of the “side population” derived from primary trophoblast cells^43^ revealed an upregulation of decorin indicating that DCN might play a role in maintaining trophoblast stem-ness. Present study reveals a novel role of DCN in trophoblast stem cell renewal and differentiation. We found that DCN restrained self-renewal of ES cells and promoted ES → TS conversion and TS cell stem-ness. Observed DCN effects on differentiation pathways using HTR cells, p-trophoblast cells and Bewo cells clearly reveal that DCN restrains TS cell differentiation to both EVT and villous (syncytial) pathways (Schema presented in Fig. 9).

Currently it remains unknown whether the observed stem-cell regulatory DCN functions play any role in the pathogenesis of PE/FGR. Recent immuno-histological studies by Weber *et al*.^36^ identified distinct trophoblast stem cell and pluripotency marker staining patterns in the human placentas depending on gestational age and placenta-associated pregnancy complications. They speculated that aberrant trophoblast stem cell differentiation may be associated with FGR. Since certain forms of FGR share the same placental pathology with PE, we speculate that DCN overproduction in the placenta/decidua may result in poor trophoblast differentiation in a subset of FGR. In a recent study we found that DCN overproduction by the decidua was causally associated with PE and elevated DCN level in maternal blood predates PE, serving as a potentially predictive biomarker for PE^32^. Elevated DCN in the maternal blood during the third trimester was reported in subjects with FGR offspring^37^.

In summary, we describe here for the first time a unique model to study trophoblast stem cell self-renewal and differentiation. Growing cells as spheroids from single cells induces the expression of both ES and TS-like stem cell markers in both HTR-8/SVneo cell line and first trimester primary trophoblast cells. This model also allows the primary trophoblast cells to proliferate for at least two weeks after isolation in contrast to monolayer culture which fail to proliferate but differentiate into syncytiotrophoblast. DCN was shown to restrain self-renewal of ES-like cells and promote ES differentiation/commitment to TS-like cells. DCN also inhibited differentiation of TS cells to villous and EVT pathways. Further studies are needed to elucidate the molecular mechanisms in DCN regulation of human trophoblast stem cell self-renewal and differentiation. It was reported that DCN retained nephron progenitor cells in an undifferentiated state through inhibition of BMP-7 signaling^34^. The role of BMP signaling in DCN mediated retention of TS cell state deserves investigation.

## Methods

HTR8/SVneo cells (referred to as HTR cells throughout the text) is a SV40Tag immortalized first trimester human trophoblast cell line^18^. As opposed to choriocarcinoma cells they are non-tumorigenic when transplanted under the kidney capsule of nude mice^44^. These cells express HLA-G mRNA. HLA-G protein expression is stimulated in the presence of laminin or matrigel^39^.

### Collection of human chorionic villi

First trimester placental tissues were obtained with written informed consent from patients undergoing elective termination of pregnancy at the London Health Sciences Centre, London, ON, Canada, following approval by the Research Ethics Board, UWO, London, ON.

### Isolation of primary cytotrophoblast cells from first trimester chorionic villi

Primary cytotrophoblast cells (henceforth referred to as p-trophoblast) were isolated using a modification of Kliman protocol^45^. First trimester villous tissue (6–9 weeks gestation) was washed in HBSS with Ca/Mg and HBSS without Ca/Mg respectively to remove all maternal blood. Villi were dissected from the membranes, transferred to 250 ml conical flask and incubated with 50 ml digestion cocktail (0.25% trypsin & 10 mg/ml DNAse I (Sigma) in HBBS per 5 g of tissue in a 37  °C water-bath for 30 min. 50 ml of wash buffer (PBS containing 2%FBS) was added before collection to wash and dilute enzymes. Tissues were then filtered through a cell dissociation sieve (Sigma CD-1, 200 mesh) and the supernatant (containing syncytium) was discarded. Additional 3 sequential digestions of the tissues (20 min each) were performed at 37 °C in shaking bath in 50 mL of Trypsin digestion mixture. Supernatants 1, 2 and 3 were collected in four 50 mL tubes with 10% FCS each time. Remaining tissue was washed with 50 mL of HBSS and the resulting supernatant was added to the 4 tubes. This represented the cytotrophoblast fraction with contaminating leukocytes and fibroblasts. Supernatants were then centrifuged for 5 min at 1500 rpm (RT), each pellets were re-suspended in 2 mL of wash buffer. Next the cells were passed through 70 µm cell strainer (BD) to get single cell suspension which were then put on top of Ficoll and centrifuged for 10 mins at 2000 rpm without a brake. Ring fractions from the middle of the tubes were collected in new tubes, one volume of wash buffer was added and centrifuged for 5 min at 1500 rpm. Pellets were re-suspended in 2 mL of wash buffer, cell number was counted and they were blocked with 1 mL MACS buffer (PBS + 0.25% BSA + 2 mM EDTA) + 10% FCS + 10% human serum for 5 min. Blocking was followed by centrifugation for 5 min at 1500 rpm and resuspension of cells in MACS buffer containing anti-fibroblast and anti-CD45 coated microbeads (60 ul MACS buffer + 20 µl anti-fibroblast + 20 µl anti-CD45 microbeads for 10^7^ cells). After Incubation for 30 min at RT 5 mL MACS buffer was added and again centrifuged for 5 min at 1500 rpm. Resulting pellet was suspended in 3 mL of MACS buffer and passed through LS 50 Macs column loaded to the magnet. Finally the fibroblast/CD45 negative fraction containing cytotrophoblast cells were collected and centrifuged for 1500 rpm 5 min and suspended in DMEM/F12 medium. Cell viability was assessed with trypan blue staining. Resulting trophoblast purity was 95–98% (identified by CK7 staining) and cell viability >99% in the final preparations.

### Spheroid formation assay

HTR8 and p-cytotrophoblast cells (within 24 hrs of isolation) were used for spheroid assay. Cells were passed through 27 gauge needle and 40 µm cell strainer to get single cell suspension. They were plated either on Ultra-low attachment 96 well (5 cells/well) plate or 6 well plate (3000 cells/well) and allowed to grow until day14 either in presence or absence of 250 nM DCN. This concentration was based on a pilot experiment using a range of 50 nM to 400 nM showing a maximal inhibitory effect on spheroid formation between 200 nM and 300 nM. Spheroid numbers and sizes were measured every 3–4 days. Spheroids were collected and used either for RNA extraction or immunofluorescence study to identify ES and TS cell markers. For some experiments, cells were isolated from spheroids (by trypsin treatment) and again used for a second generation spheroid assay or plated on GFR matrigel coated plate/coverslips for subsequent analysis of EVT pathway markers.

### RNA extraction/cDNA preparation/qRT-PCR

RNA was extracted using RNeasy Mini Kit (Qiagen) according to the manufacturer’s protocol. cDNA was synthesized with ~500 ng of RNA using High-Capacity cDNA Reverse Transcription Kit (Applied Biosystem). qRT-PCR was run on a LightCycler (Bio-Rad) using custom designed primers and SYBR Green PCR mix (Quanta Bioscience). ΔΔCt method was employed to determine mRNA expression relative to β-actin/GAPDH/18 S rRNA for each sample.

### Immunofluorescence staining of trophoblast spheroids

Trophoblast spheroids were carefully pipetted in a microfuge tube and allowed to settle down (~15 mins). Any excess medium was removed from spheroids, washed for 3 times with PBS and 200 uL of 4% PFA was added for 30 mins. PFA was removed and spheroids were washed 3 times with PBS. Next, 0.5% Triton X-100 was added to permeabilize them for 10 mins and washed with 200 ul PBS 3 times. They were blocked with 500 uL of 8% BSA in PBS with 0.01% Tween20 for 30 mins and washed 3 times with 200 uL PBS. Primary antibody at recommended dilution in 8% BSA-PBS were used and incubated overnight in 4 °C. Next day, they were washed twice with PBS and fluorescent tagged secondary antibody was added and incubated for 1 hr at room temperature. Next, secondary antibody was removed, washed with PBS and 20 ul of mounting media (Vectashield Mounting medium for fluourescence with DAPI H-1200; Vector Laboratories) was added for 5 mins. Then 10 uL was added onto each slide (Fisherbrand Colorfrost Plus Microscope Slides 25 × 75 × 1.0 mm #12-550-20), coverslip was put on and slides were dried horizontally in 4 °C overnight. Next day a clear nailpolish was used to seal the ends of the coverslip and stored at 4 °C until used for confocal imaging.

### Statistical analysis

Statistical calculations were performed using GraphPad Prism software version 5 (GraphPad Software, CA). All parametric data were analyzed with one-way ANOVA followed by Tukey-Kramer or Dunnett post-hoc comparisons. Student’s t-test was used when comparing two datasets. Significant differences between means were accepted at p < 0.05

### Data availability statement

All data generated or analyzed during this study are included in this published article.

### Experimental methods guideline statement

All experiments were performed in accordance with relevant guidelines and regulations.

## Electronic supplementary material

Supplementary Information

## References

[1] Tanaka S, Kunath T, Hadjantonakis AK, Nagy A, Rossant J (1998). Promotion of Trophoblast stem cell proliferation by FGF4. Science.

[2] Rossant, J. Stem cells and lineage development in the mammalian blastocyst. *Reproduction, Fertility and Development* 19, 10.1071/rd06125 (2007). 10.1071/rd06125 17389140

[3] Genbacev O (2011). Establishment of human trophoblast progenitor cell lines from the chorion. Stem Cells.

[4] Xu RH (2002). BMP4 initiates human embryonic stem cell differentiation to trophoblast. Nat. Biotechnol..

[5] Golos TG, Giakoumopoulos M, Gerami-Naini B (2013). Review: Trophoblast differentiation from human embryonic stem cells. Placenta..

[6] Li Y, Parast MM (2014). BMP4 regulation of human trophoblast development. Int. J. Dev. Biol..

[7] Yabea, S. *et al*. Comparison of syncytiotrophoblast generated from human embryonic stem cells and from term placentas. *Proc. Natl. Acad. Sci. USA* 113, 10.1073/pnas.1601630113 (2016). 10.1073/pnas.1601630113 PMC4868474 27051068

[8] Hemberger M, Udayashankar R, Tesar P, Moore H, Burton GJ (2010). ELF5-enforced transcriptional networks define an epigenetically regulated trophoblast stem cell compartment in the human placenta. Hum. Mol. Genet..

[9] Chang CW, Parast MM (2017). Human trophoblast stem cells: Real or not real?. Placenta.

[10] Okae H (2018). Derivation of Human Trophoblast Stem Cells. Cell Stem Cell.

[11] James, J. L., Carter, A. M. & Chamley, L. W. Human placentation from nidation to 5 weeks of gestation. Part I: What do we know about formative placental development following implantation? *Placenta* 33, 10.1016/j.placenta.2012.01.020 (2012). 10.1016/j.placenta.2012.01.020 22374510

[12] Lala PK, Chakraborty C (2003). Factors Regulating Trophoblast Migration and Invasiveness: Possible Derangements Contributing to Pre-eclampsia and Fetal Injury. Placenta.

[13] Burton GJ, Woods AW, Jauniaux E, Kingdom JCP (2009). Rheological and Physiological Consequences of Conversion of the Maternal Spiral Arteries for Uteroplacental Blood Flow during Human Pregnancy. Placenta.

[14] Damsky, C. H. *et al*. Integrin switching regulates normal trophoblast invasion. *Development* 120 (1994). 10.1242/dev.120.12.3657 7529679

[15] Zhou Y, Damsky CH, Chiu K, Roberts JM, Fisher SJ (1993). Preeclampsia is associated with abnormal expression of adhesion molecules by invasive cytotrophoblasts. J. Clin. Invest..

[16] Horii M (2016). Human pluripotent stem cells as a model of trophoblast differentiation in both normal development and disease. Proc. Natl. Acad. Sci. USA.

[17] Slack, J. M. W. Stem Cells in Epithelial Tissues. *Science* 287, 10.1126/science.287.5457.1431 (2000). 10.1126/science.287.5457.1431 10688782

[18] Graham C (1993). Establishment and Characterization of First Trimester Human Trophoblast Cells with Extended Lifespan. Experimental Cell Research.

[19] Cloutier-Bosworth, A. The Role of Nodal in the Regulation of Bi-Potential Trophoblast Progenitor Cells. *Master of Science thesis, Western University* (2012).

[20] Takao T (2011). Isolation and Characterization of Human Trophoblast Side-Population (SP) Cells in Primary Villous Cytotrophoblasts and HTR-8/SVneo Cell Line. PLoS ONE.

[21] Weber M, Knoefler I, Schleussner E, Markert UR, Fitzgerald JS (2013). HTR8/SVneo cells display trophoblast progenitor cell-like characteristics indicative of self-renewal, repopulation activity, and expression of “stemness-“ associated transcription factors. Biomed. Res. Int..

[22] Reynolds B, Weiss S (1992). Generation of neurons and astrocytes from isolated cells of the adult mammalian central nervous system. Science.

[23] Dontu G, Al-Hajj M, Abdallah WM, Clarke MF, Wicha MS (2003). Stem cells in normal breast development and breast cancer. Cell Prolif..

[24] Shaw FL (2012). A detailed mammosphere assay protocol for the quantification of breast stem cell activity. J. Mammary Gland Biol. Neoplasia.

[25] Majumder M (2016). COX-2 Induces Breast Cancer Stem Cells via EP4/PI3K/AKT/NOTCH/WNT Axis. Stem Cells.

[26] Iozzo RV (1998). Matrix proteoglycans: from molecular design to cellular function. Annu. Rev. Biochem..

[27] Lysiak JJ, Hunt J, Pringle GA, Lala PK (1995). Localization of transforming growth factor β and its natural inhibitor decorin in the human placenta and decidua throughout gestation. Placenta.

[28] Xu G, Guimond MJ, Chakraborty C, Lala PK (2002). Control of proliferation, migration, and invasiveness of human extravillous trophoblast by decorin, a decidual product. Biol. Reprod..

[29] Iacob D (2008). Decorin-mediated inhibition of proliferation and migration of the human trophoblast via different tyrosine kinase receptors. Endocrinology.

[30] Khan GA, Girish GV, Lala N, Di Guglielmo GM, Lala PK (2011). Decorin is a novel VEGFR-2-binding antagonist for the human extravillous trophoblast. Mol. Endocrinol..

[31] Lala N, Girish GV, Cloutier-Bosworth A, Lala PK (2012). Mechanisms in decorin regulation of vascular endothelial growth factor-induced human trophoblast migration and acquisition of endothelial phenotype. Biol. Reprod..

[32] Siddiqui MF (2016). Decorin over-expression by decidual cells in preeclampsia: a potential blood biomarker. Am. J. Obstet. Gynecol..

[33] Lala PK, Nandi P (2016). Mechanisms of trophoblast migration, endometrial angiogenesis in preeclampsia: The role of decorin. Cell Adh. Migr..

[34] Fetting JL (2014). FOXD1 promotes nephron progenitor differentiation by repressing decorin in the embryonic kidney. Development.

[35] Chen XD, Dusevich V, Feng JQ, Manolagas SC, Jilka RL (2007). Extracellular matrix made by bone marrow cells facilitates expansion of marrow-derived mesenchymal progenitor cells and prevents their differentiation into osteoblasts. J. Bone Miner. Res..

[36] Weber M (2016). Unique trophoblast stem cell- and pluripotency marker staining patterns depending on gestational age and placenta-associated pregnancy complications. Cell Adh. Migr..

[37] Caglar M (2014). Decorin: a possible marker for fetal growth restriction. Gynecol. Endocrinol..

[38] McMaster M (1998). HLA-G Isoforms Produced by Placental Cytotrophoblasts and Found in Amniotic Fluid Are Due to Unusual Glycosylation. J. Immunol..

[39] Zdravkovic M (1999). Susceptibility of MHC class I expressing extravillous trophoblast cell lines to killing by natural killer cells. Placenta.

[40] Latos PA, Hemberger M (2014). Review: the transcriptional and signalling networks of mouse trophoblast stem cells. Placenta.

[41] Janatpour MJ (2000). Id-2 regulates critical aspects of human cytotrophoblast differentiation, invasion and migration. Development.

[42] Balahmar, R. M. *et al*. Identification and characterisation of NANOG+/OCT-4high/ SOX2+ doxorubicin-resistant stem-like cells from transformed trophoblastic cell lines. *Oncotarget*10.18632/oncotarget.24151 (2018). 10.18632/oncotarget.24151 PMC5805535 29467949

[43] James JL (2015). Isolation and characterisation of a novel trophoblast side-population from first trimester placentae. Reproduction.

[44] Khoo NK (1998). SV40 Tag transformation of the normal invasive trophoblast results in a premalignant phenotype. I. Mechanisms responsible for hyperinvasiveness and resistance to anti-invasive action of TGF-beta. Int. J. Cancer.

[45] Kliman HJ (1986). Purification, characterization, and *in vitro* differentiation of cytotrophoblasts from human term placentae. Endocrinology.

