# Supplementary material for: Human trophoblast stem cell self-renewal and differentiation: Role of decorin
Source: Sci Rep. 2018 Jun 12;8:8977. doi: 10.1038/s41598-018-27119-4 (PMC5997742; doi:10.1038/s41598-018-27119-4)
Supplement: Supplementary file 1 — Supplementary Information [file 41598_2018_27119_MOESM1_ESM.pdf]

Human trophoblast stem cell self-renewal and differentiation: Role of decorin

Pinki Nandi[1], Hyobin Lim[1], Eloy Jose Torres-Garcia[1], Peeyush K Lala[1,2,3,*]

[1]Department of Anatomy and Cell Biology, Schulich School of Medicine and Dentistry, University of Western Ontario, London, Ontario, Canada.

[2] Department of Oncology, Schulich School of Medicine and Dentistry, University of Western Ontario, London, Ontario, Canada.

[3] Associate Scientist, Children's Health Research Institute, Schulich School of Medicine and Dentistry, University of Western Ontario, London, Ontario, Canada.

*Corresponding Author (Correspondence to pklala@uwo.ca)

Supplementary Figure 1

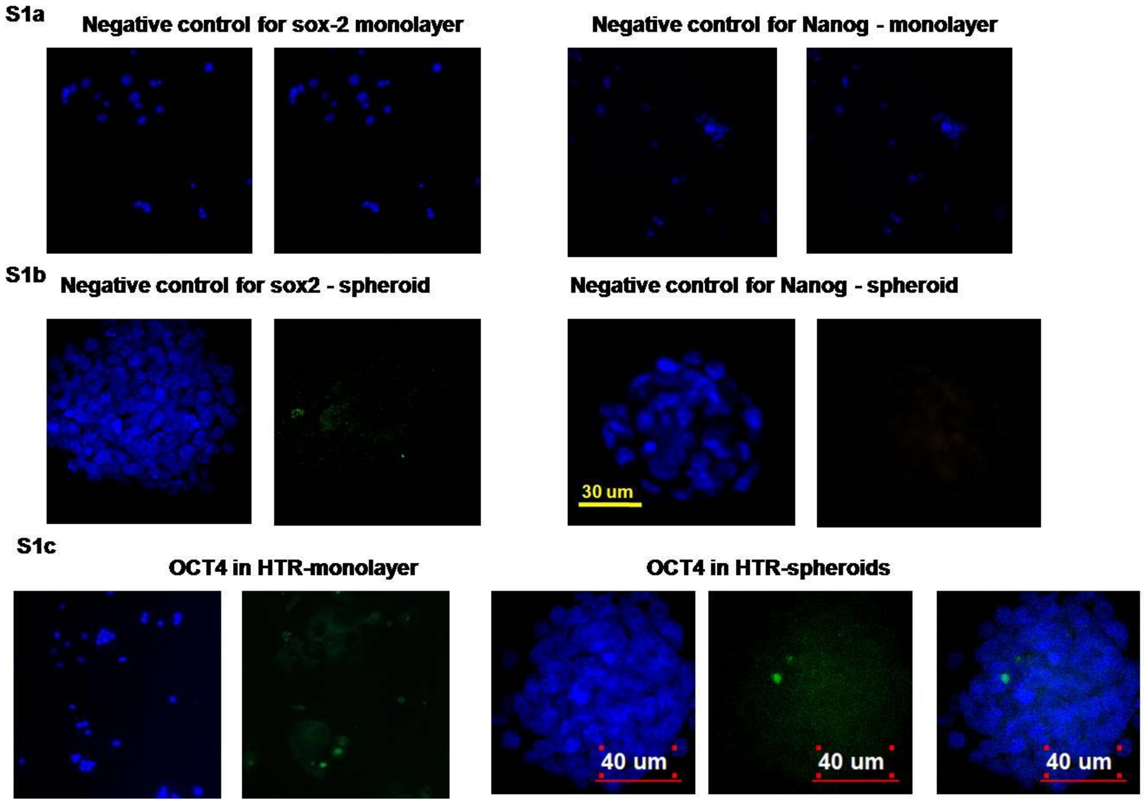

Figure legend for supplementary figure 1: S1a shows the negative controls for SOX2 (left panel) and NANOG (right panel) in HTR-monolayer. Figure S1b shows the negative controls for SOX2 (left panel) and NANOG (right panel) in HTR-spheroids. Figure S1c shows staining for OCT4 in HTR monolayer and spheroids.

Supplementary Figure 2

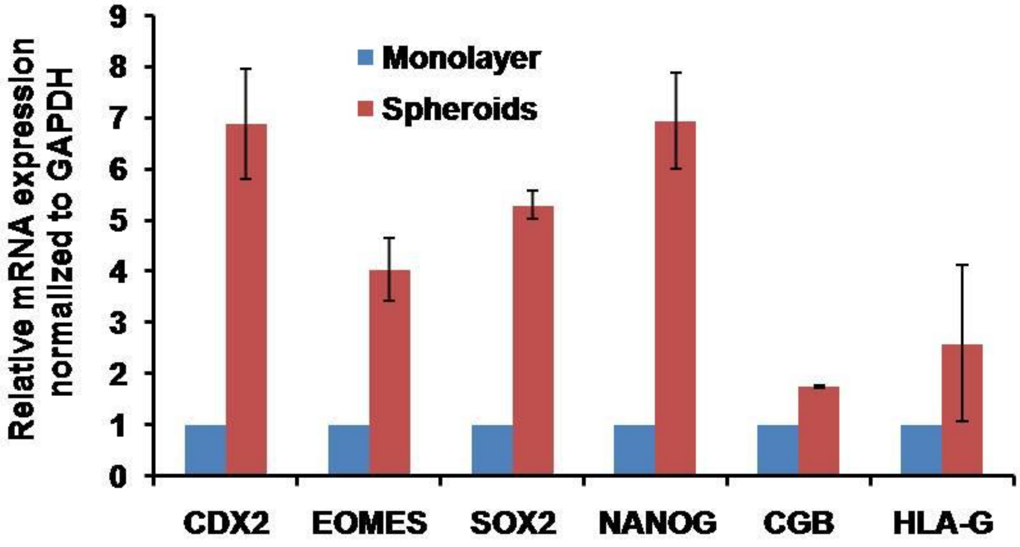

Figure legend for supplementary figure 2: qRT-PCR analysis of ES (SOX2. NANOG), TS (CDX2. EOMES) and differentiation markers (CGB and HLA-G) in a population of pooled HTR-spheroids.
